# Variation in Access to Palliative Radiotherapy in Prostate Cancer: A Population-Based Study in Canada

**DOI:** 10.7759/cureus.54582

**Published:** 2024-02-20

**Authors:** Alborz Jooya, Daniel Qureshi, William J Phillips, Jennifer Leigh, Colleen Webber, Ajay Aggarwal, Peter Tanuseputro, Scott Morgan, Robert Macrae, Michael Ong, Jean-Marc Bourque

**Affiliations:** 1 Department of Radiation Oncology, Princess Margaret Cancer Centre, University Health Network (UHN), Toronto, CAN; 2 Department of Public Health, London School of Hygiene and Tropical Medicine, London, GBR; 3 Department of Medicine, The Ottawa Hospital, Ottawa, CAN; 4 Department of Medicine, The Ottawa Hospital Research Institute, Ottawa, CAN; 5 Department of Oncology, Guy’s Cancer Centre, London, GBR; 6 Department of Radiation Oncology, University of Ottawa, Ottawa, CAN; 7 Division of Medical Oncology, Department of Internal Medicine, University of Ottawa, Ottawa, CAN; 8 Department of Radiation Oncology, Montreal University Health Center, Montreal, CAN

**Keywords:** barriers to access, utilization of radiotherapy, ontario, prostate cancer, palliative radiotherapy

## Abstract

Background: As a result of improvements in cancer therapies, patients with metastatic malignancies are living longer, and the role of palliative radiotherapy has become increasingly recognized. However, access to adequate palliative radiotherapy may continue to be a challenge, as is evident from the high proportion of patients dying of prostate cancer who never receive palliative radiotherapy. The main objective of this investigation is to identify and describe the factors associated with the receipt of palliative radiation treatment in a decedent cohort of prostate cancer patients in Ontario.

Methodology: Population-based administrative databases from Ontario, Canada, were used to identify prostate cancer decedents, 65 years or older who received androgen deprivation therapy between January 1, 2013, and December 31, 2018. Baseline and treatment characteristics were analyzed using univariate and multivariate logistic regression models for association with receipt of radiotherapy in a two-year observation period before death.

Results: We identified 3,788 prostate cancer decedents between 2013 and 2018; among these, 49.9% received radiotherapy in the two years preceding death. There were statistically significant positive associations between receipt of radiotherapy and younger age at diagnosis (odds ratio [OR] 1.6, 95% confidence interval [CI] 1.1-2.3); higher stage at diagnosis (OR 1.3, 95% CI 1.1-1.7); receipt of care at a regional cancer center (OR 1.8, 95% CI 1.3-2.4); and involvement of radiation oncologists (OR 155.1, 95% CI 83.3-288.7) or medical oncologists (OR 1.4, 95% CI 1.1-1.8). However, there were no associations between receipt of radiotherapy and income, distance to the nearest cancer center, involvement of urologists in cancer care, healthcare administrative region, home-care involvement, or number of hospitalizations in the observation period.

Conclusions: We found the utilization of palliative radiotherapy for prostate cancer patients in Ontario varies depending on age, stage at diagnosis, number of comorbidities, registration at regional cancer centers, and involvement of oncologists. There were no differences detected based on income or distance from a cancer center. The findings of this study represent an important opportunity to facilitate better access to palliative radiotherapy and referrals to multidisciplinary regional cancer centers, to improve the quality of life of this patient population.

## Introduction

Prostate cancer is the most common cancer in men, accounting for nearly 20% of all new cancer diagnoses in Canada and the United States, and it is considered a leading cause of morbidity and mortality [1,2]. As a result of improvements in systemic therapy, patients with metastatic malignancies are living longer [3], and nearly half of all the courses of radiotherapy are with palliative intent [4,5]. There is a well-recognized role for palliative radiation therapy following prostate-directed treatments, especially in a subset of patients with de novo metastases or ones who develop resistance to androgen deprivation therapy (ADT), with resultant detriment to quality of life mostly due to the increasing burden of metastatic disease.

Disparities in the receipt of cancer care and supportive care services in countries where cancer care is publicly insured have been linked to socioeconomic deprivation [6], income level, and geographical area and have been associated with poor survival among patients with prostate cancer [7-11].

In a recent study that identified social and health system-related factors affecting the use of palliative radiotherapy to define a benchmark population of patients with unimpeded access to radiation therapy in the province of Ontario, 33.9% of patients with no access limitation who died of any type of cancer had received palliative radiation therapy, compared to only 28.5% of all adult patients who died of cancer [12]. Although not specific to prostate cancer, the difference in rates of receipt of palliative radiotherapy between patient populations without barriers to access compared with all patients points to potential gaps in access to radiation treatment services that could explored to improve the quality of life of many metastatic patients.

We believe that access to advanced prostate cancer therapies is not uniform across Ontario, and there may be many factors contributing to this disparity. To improve access and equity among patients, it is imperative to gain an understanding of the patterns of use of palliative radiotherapy in prostate cancer patients in Ontario and assess the underlying factors associated with disparities in care. Thus, we sought to (1) describe the utilization of radiotherapy among prostate cancer decedents who died between 2013 and 2018 in Ontario and (2) identify factors associated with variation in access to palliative radiation treatment.

## Materials and methods

Identification of the cohort

We conducted a retrospective cohort study using population-based administrative databases held at ICES (previously known as the Institute for Clinical Evaluative Sciences). ICES is an independent, nonprofit research institute whose legal status under Ontario’s health information privacy law allows it to collect and analyze healthcare and demographic data, without consent, for health system evaluation and improvement.

We identified all patients aged 65 years or older who would be eligible for the Ontario Drug Benefit (ODB) Program, with a diagnosis of prostate cancer in the Ontario Cancer Registry between January 1, 2007, and December 31, 2018. Our main analysis was focused on the last two years before death (index date) to determine the receipt of radiotherapy about patient and disease factors in the palliative setting. We narrowed our cohort to patients who received ADT or had a bilateral orchiectomy after diagnosis, and who died of prostate cancer in the period from January 1, 2013, and December 31, 2018, because of the adoption of androgen-receptor axis-targeted therapies and subsequent change in medical management of patients with mostly metastatic prostate cancer, who would be eligible for novel androgen targeted therapies and palliative radiotherapy.

Data sources

Multiple administrative databases were linked to obtain patients’ data from the index date, defined as the date two years before death. These datasets were linked using unique encoded identifiers and analyzed at ICES. The authors do not own these data and, hence, are not permitted to share them in the original form (only in aggregate form). The databases utilized included Ontario Drug Benefit, Ontario Cancer Registry, and Activity-Level Reporting (ALR), which captures activity within the cancer system, including receipt of systemic therapies and radiotherapy. Descriptions of the information they provided can be found in Appendix A. Use of the data in this project was authorized under Section 45 of Ontario’s Personal Health Information Protection Act (PHIPA) and did not require review by a Research Ethics Board.

Characteristics of interest

Sociodemographic data, including age, area-level income quintile (dividing the population in a neighborhood into five income groups from lowest to highest income), Ontario Local Health Integration Network (LHIN) region (providing healthcare services to their respective regions), count of chronic diseases (including respiratory, cardiovascular, renal, etc.), Charlson Comorbidity Index Score (CCI), home care registration, distance to a cancer center, registration at a regional cancer center at diagnosis, and long-term care (LTC) residency were collected at the study index date. Physician encounters, including the number of visits, within the two-year study period were captured from the Ontario Health Insurance Program (OHIP) database, and physician specialty was obtained from the ICES Physician Database (IPDB). Other variables in the two-year lookback period before death that were collected included the number of hospitalizations, type of oncology specialists involved (urologists, medical oncologists, and radiation oncologists), and availability of family practitioners (identified from OHIP billings). Disease-specific characteristics were obtained from the Ontario Cancer Registry (OCR) and included TNM stage at diagnosis, year of diagnosis, castrate resistance status, and Gleason score. Prostate-specific antigen (PSA) at ADT initiation was captured from the Ontario Laboratories Information System (OLIS). Receipt of systemic therapies, including life-prolonging therapies (LPTs), such as abiraterone, enzalutamide, docetaxel, cabazitaxel, radium-223, and bone-targeting agents, such as denosumab and zoledronate, and their use within the study period were captured using the ODB and New Drug Funding Program (NDFP) databases.

Outcomes of interest

For analysis, baseline and treatment characteristics were analyzed for association with receipt of radiotherapy in a two-year observation period before death. The primary outcome included factors associated with access to any radiotherapy, radiotherapy to bone, and radiotherapy to the prostate. Radiotherapy information was captured using the Cancer ALR database, which includes data on patient-level activity within the cancer system. ALR collects selected systemic therapy and all radiation treatment information, including dose fractionation schemes, from regional cancer centers and their associated hospitals; although not specifically for prostate cancer, it has been validated for use in cancer research [13].

Statistical analysis

Descriptive statistics were used to describe patient and disease characteristics as categorized by receipt of any radiotherapy versus none and radiotherapy to bone versus none. Univariate and multivariate logistic regression models were used to assess the factors associated with receipt of any radiotherapy. Analysis of Maximum Likelihood Estimates and Wald Chi-Square were utilized to assess whether a variable was statistically significant or not. The adjusted variables included age, stage at diagnosis, year of diagnosis, count of chronic diseases, CCI, area-level income quintile, LHIN region, distance to cancer center, involvement of urologists, medical or radiation oncologists, regional cancer center registration at diagnosis, home care involvement, LTC residency, number of hospitalizations, and prior prostate directed therapy or systemic therapy. Moreover, trends in receipt of radiotherapy at the time of death were examined. To avoid the potential bias from doing a complete case analysis, the regression models were performed using the available data. Specifically, the absence of data on PSA and stage at diagnosis for patients diagnosed before 2007 skewed the multivariable analyses in favor of patients diagnosed after 2007. Moreover, given the high numbers of missing data for PSA and Gleason scores, these variables were not included in the multivariate analysis. Statistical significance was defined as *P* ≤ 0.05. All analyses were conducted using SAS Enterprise Guide 7.1 (SAS Institute Inc., Cary, NC).

## Results

Receipt of radiotherapy

We identified 3,788 patients in Ontario, Canada, with the diagnosis of prostate cancer who received ADT and who were identified to have had a prostate-cancer-related death between 2013 and 2018 (Appendix B). Of the 3,788 included patients, 1,890 (49.9%) received radiotherapy during the two-year observation period.

Of all the patients who received radiation, 1,724 (91.2%) underwent palliative radiotherapy to a bony site and 186 (4.9%) received prostate-directed radiotherapy (of which, 86, 46.2%, had curative and 103, 55.4%, had palliative intent of prostate treatment). Receipt of radiotherapy is further subclassified below based on several characteristics within the cohort.

Sociodemographic Characteristics

The mean age at initiating ADT was 75.5 years for patients who received radiotherapy and 79.6 years for those who did not. In the lowest age category of 65-69 years, 516 (67.4%) received any radiotherapy, compared to 264 (32.7%) of those aged 85 or older who underwent radiation treatment. This was in contrast to 250 (32.6%) and 543 (67.3%) patients who did not receive RT who were in the 65-69 and 85+ age categories, respectively (Table 1). The proportion of patients with the highest area-level income quintile (*n *= 414, 53.8%, and *n *= 355, 46.2%) was similar to the patients in the lowest income bracket (*n *= 371, 49.7%, and *n* = 376, 50.3%) for patients who underwent radiotherapy and ones who did not, respectively.

**Table 1:** Patient characteristics of prostate cancer decedents. Patient characteristics are stratified by whether radiotherapy was received during the two-year observation period.

Characteristic	Description	Any radiotherapy, *n* (%) (*n *= 1,890, 49.9%)	No radiotherapy, *n* (%) (*n *= 1,898, 50.1%)	Overall (*n* = 3,788)
Age (years)	65-69	516 (67.4%)	250 (32.6%)	766
	70-74	386 (58.7%)	272 (41.3%)	658
	75-79	387 (52.3%)	353 (47.7%)	740
	80-84	337 (41.2%)	480 (58.8%)	817
	85+	264 (32.7%)	543 (67.3%)	807
Year of diagnosis	2007-2011	727 (57.0)	809 (43.0)	1,536
	2012-2017	1,083 (70.8)	946 (29.2)	2,029
Area-level income quintile	1 (lowest)	371 (49.7%)	376 (50.3%)	747
	2	366 (49.5%)	373 (50.5%)	739
	3	370 (47.9%)	402 (52.1%)	772
	4	369 (48.5%)	392 (51.5%)	754
	5 (highest)	414 (53.8%)	355 (46.2%)	769
Distance to cancer center (km)	Mean (SD)	34.7 (52.2)	36.7 (51.6)	35.7 (51.9)
Charlson Comorbidity Index Score	≤2	1,644 (50.5%)	1,613 (49.5%)	3,257
	3-4	89 (42.2%)	122 (57.8%)	211
	≥5	157 (49.1%)	163 (50.9%)	320
Count of chronic diseases	Mean (SD)	3.30 (1.9)	3.91 (2.1)	3.6 (2.0)
Physicians involved in cancer care	Radiation oncologist	1,877 (72.1%)	725 (27.9%)	2,602
	Medical oncologist	1,577 (58.9%)	1,101 (41.1%)	2,678
	Urologist	1,601 (49.7%)	1,623 (50.3%)	3,224
Mean/Median number of visits by physician specialty (SD)	Radiation oncologist	7.38 (6.0)/6 (3-10)	1.37 (3.1)/0 (0-2)	4.4 (4.5)
	Medical oncologist	19.6 (24.3)/11 (2-29)	10.5 (20.5)/1 (0-11)	15.0 (22.4)
	Urologist	8.6 (8.3)/7 (2-12)	8.8 (9.3)/7 (3-12)	8.7 (8.8)
	Family physician	54.0 (35.2)/47 (30-69)	46.8 (35.1)/38 (23-59)	50.4 (35.1)
Rostered to primary care	Yes	1,576 (49.8%)	1,587 (50.2%)	3,163
	No	314 (50.2%)	311 (49.8%)	625
Consultation at Regional Cancer Center	Yes	1,625 (63.8%)	922 (36.2%)	2,547
	No	265 (21.4%)	976 (78.6%)	1,241
Long-term care residency	Yes	6 (8.8%)	62 (91.2%)	68
	No	1,884 (50.6%)	1,836 (49.4%)	3,720
Home care involvement	Yes	227 (39.3%)	351 (60.7%)	578
	No	1,663 (51.8%)	1,547 (48.2%)	3,210

Receipt of radiotherapy across Ontario’s LHINs ranged from 38.7% to 63.6% (Table 2).

**Table 2:** LHIN region of prostate cancer decedents stratified by whether radiotherapy was received. LHIN, Local Health Integration Network

LHIN region	Any radiotherapy, *n* (%) (*n *= 1,890, 49.9%)	No radiotherapy, *n* (%) (*n *= 1,898, 50.1%)	Overall (*n *= 3,788)	Univariate odds ratio (95% CI)	Multivariate *P*-value
8 - *n* (%)	187 (47.5%)	207 (52.5%)	394	Reference	0.12
9 - *n* (%)	184 (42.8%)	246 (57.2%)	430	0.72 (0.47-1.10)	0.67
5 - *n* (%)	83 (47.2%)	93 (52.8%)	176	1.14 (0.62-2.09)	0.13
11 - *n* (%)	225 (63.6%)	129 (36.4%)	354	1.43 (0.90-2.25)	0.71
1 - *n* (%)	91 (50.6%)	89 (49.4%)	180	0.90 (0.51-1.57)	0.59
4 - *n* (%)	221 (47.7%)	242 (52.3%)	463	1.12 (0.73-1.72)	0.42
6 - *n* (%)	135 (50.8%)	131 (49.2%)	266	1.22 (0.75-1.99)	0.08
13 - *n* (%)	119 (53.1%)	105 (46.9%)	224	0.64 (0.39-1.05)	0.13
12 - *n* (%)	60 (38.7%)	95 (61.3%)	155	0.63 (0.35-1.15)	0.03
14 - n (%)	43 (52.4%)	39 (47.6%)	82	0.49 (0.26-0.93)	0.62
10 - *n* (%)	125 (54.6%)	104 (45.4%)	229	1.14 (0.67-1.95)	0.79
2 - *n* (%)	150 (47.5%)	166 (52.5%)	316	1.06 (0.67-1.68)	0.76
7 - *n* (%)	140 (49.0%)	146 (51.0%)	286	1.08 (0.65-1.79)	0.92
3 - *n* (%)	127 (54.5%)	106 (45.5%)	233s	1.03 (0.62-1.69)	0.12

Also, the mean distance to the nearest cancer center from the resident’s home was 34.7 and 36.7 km for patients who received any radiotherapy and those who did not, respectively, with equivalent proportions receiving radiation treatments irrespective of the distance to the nearest cancer care center.

The majority of patients (3,257, 85.9%) had a CCI score of 2 or less, while 320 (8.4%) had a score of 5 or greater. Among patients who received radiotherapy, 1,644 (50.5%) patients had a CCI score of 2 or less versus 1,613 (49.5%) patients who did not receive radiation treatments.

Healthcare Services

Among patients who received care from specialist physicians, including medical oncologists (*n *= 2,678, 70.7%), radiation oncologists (*n *= 2,602, 68.7%), and urologists (*n *= 3,224, 85.1%), 58.9%, 72.1%, and 49.7% received any radiotherapy, respectively. Although other specialties may have been involved in patients' care, the analysis focused on these specialties, as they constitute the primary services involved in the management of patients with prostate cancer. For patients receiving radiotherapy, the mean number of encounters was 19.6 medical oncology visits, 7.4 radiation oncology visits, and 8.6 urology visits. For patients who did not receive radiotherapy, the mean number of encounters was 10.5 medical oncology visits, 1.37 radiation oncology visits, and 8.79 urology visits. A total of 3,163 (83.5%) were rostered to primary care, of which 1,576 (49.8%) received radiation. Among 625 (16.5%) not rostered to primary care, 314 (50.2%) received radiotherapy. Of the 2,547 (67.2%) patients who were registered at a regional cancer center, 1,625 (63.8%) received radiation services. In contrast, only 265 (21.4%) of 1,241 patients were not registered to receive radiotherapy. Of 68 (1.8%) patients who were LTC residents, a minority (*n *= 6, 8.8%) received radiotherapy. Furthermore, of 578 (15.2%) patients who had home care services, 227 (39.3%) underwent radiation treatments.

Disease-Specific Characteristics

Disease-specific characteristics are outlined in Table 3.

**Table 3:** Disease characteristics of prostate cancer decedents. Patient characteristics are stratified by whether radiotherapy was received. The stage at diagnosis and M status definitions are based on AJCC 6th edition definitions. AJCC, American Joint Committee on Cancer; LPT, life-prolonging therapies; PSA, prostate-specific antigen; ADT, androgen deprivation therapy

Characteristic	Description	Any radiotherapy, *n* (%) (*n *= 1,890, 49.9%)	No radiotherapy, *n* (%) (*n *= 1,898, 50.1%)	Overall (*n *= 3,788)
Stage at diagnosis	I/II/III	672 (47.0%)	758 (53.0%)	1,430
	IV	1,071 (56.8%)	816 (43.2%)	1,887
	Missing	147 (31.2%)	324 (68.8%)	471
M category at diagnosis	M0	572 (46.4%)	661 (53.6%)	1,233
	M1	853 (57.4%)	632 (42.6%)	1,485
	Missing	465 (43.5%)	605 (56.5%)	1,070
PSA at first ADT initiation (ng/mL)	<10	139 (51.1%)	133 (48.9%)	272
	11-19	118 (50.4%)	116 (49.6%)	234
	20-99	298 (52.8%)	266 (47.2%)	564
	100-1,000	285 (53.7%)	246 (46.3%)	531
	>1,000	76 (53.1%)	67 (46.9%)	143
	Missing	974 (47.7%)	1,070 (52.3%)	2,044
Orchiectomy	Yes	19 (39.6%)	29 (60.4%)	43
	No	1,871 (50.0%)	1,869 (50.0%)	3,740
Castrate resistant	Yes	1,291 (54.7%)	1,068 (45.3%)	2,359
	No	509 (41.3%)	724 (58.7%)	1,233
	Missing	90 (45.9%)	106 (54.1%)	196
Prostatectomy after diagnosis	Yes	122 (55.7%)	97 (44.3%)	219
	No	1,768 (49.5%)	1,801 (50.5%)	3,569
Systemic therapy	LPT + bone-targeted Tx	643 (63.8%)	365 (36.2%)	1,008
	LPT alone	468 (61.5%)	293 (38.5%)	761
	Bone-targeted Tx alone	156 (61.2%)	99 (38.8%)	255
	None	623 (35.3%)	1,141 (64.7%)	1,764
Radiotherapy as inpatient	Yes	149 (47.6)	164 (52.4)	313
	No	1,983 (60.8)	1,279 (39.2)	3,262

A total of 1,536 patients (40.5%) were diagnosed between 2007 and 2011, and 2,252 patients (59.4%) were diagnosed between 2012 and 2018, with 47.3% and 51.6% receiving radiation, respectively. Of the patients with stages I, II, and III (*n *= 1,430) at diagnosis who later developed stage IV disease and died of prostate cancer, 672 (47%) received radiotherapy. Additionally, among patients diagnosed with stage IV at the outset (*n* = 1,887), 1,071 (56.8%) received radiotherapy. A total of 2,359 patients (62.3%) were found to have castrate-resistant prostate cancer, with 1,291 (54.7%) of them undergoing radiation treatments. Among the 2,024 patients (53.4% of all) who received LPT or bone-targeted therapies or both, 1,267 (62.6%) also received radiotherapy. In contrast, 623 (35.3%) of the 1,764 patients who had not received any systemic treatments other than ADT underwent radiotherapy. Although only a very small proportion of all patients underwent orchiectomy, 39.6% of them received radiotherapy; this is in contrast to 50.0% of those who did not undergo orchiectomy. Also, only 219 patients had prostatectomy after diagnosis, but 55.7% of them received any radiotherapy compared with 49.5% of patients who did not undergo the procedure

Receipt of systemic therapy

We identified 2,024 patients (53.4%) in the cohort who received LPT (*n *= 1769) or bone-targeted therapy (*n *= 1,263) during the study period. Of those, 761 (37.6%) and 255 (12.6%) received LPT and bone-targeted treatments alone, respectively. The type of LPT received included abiraterone (*n *= 1,094, 61.8%), docetaxel (*n *= 881, 49.8%), enzalutamide (*n *= 480, 27.1%), radium-223 (*n *= 250, 14.1%), and cabazitaxel (*n *= 108, 6.1%). The type of bone-targeted therapy received included denosumab (*n *= 1,045, 82.7%) and zoledronate (*n *= 314, 24.8%).

A total of 901 (23.8%) patients received prostate-directed therapy after the diagnosis, with 219 (5.8%) undergoing prostatectomy and 189 (4.9%) receiving radiotherapy to the prostate.

Multivariate relationships between the receipt of radiotherapy and patient characteristics

Results of the multivariate logistic regression analyses for receipt of radiotherapy are outlined in Table 4.

**Table 4:** Univariable and multivariable logistic regression analyses of population characteristics and association with radiotherapy use. CI, confidence interval; LPT, life-prolonging therapy

Characteristics		Univariate *P*-value	Multivariate odds ratio (95% CI)	Multivariate *P*-value (chi-square)
Age (Years)	65-69	<0.0001	Reference	
	70-74		1.59 (1.12-2.27)	0.01
	75-79		1.28 (0.91-1.82)	0.16
	80-84		1.01 (0.73-1.41)	0.95
	85+		Reference	
Income	Quartile 1 (Lowest)	0.25	1.11 (0.81-1.53)	0.51
	Quartile 2		0.99 (0.73-1.35)	0.97
	Quartile 3		0.98 (0.72-1.32)	0.87
	Quartile 4		0.84 (0.62-1.13)	0.24
	Quartile 5 (Highest)		Reference	
Count of chronic diseases	0-2		Reference	
	3-4		0.92 (0.73-1.16)	0.47
	5+		0.77 (0.60-0.99)	0.04
Physicians involved in their cancer care	Radiation oncologist	<0.0001	155.07 (83.29-288.72)	<0.0001
	Medical oncologist	<0.0001	1.42 (1.09-1.84)	0.01
	Urologist	0.48	1.20 (0.92-1.56)	0.18
Registration at the Regional Cancer Center	Yes	<0.0001	1.77 (1.29-2.43)	0.0004
	No		Reference	
Distance to cancer center (km)	0-5.9		Reference	
	6-15.9		1.05 (0.79-1.39)	0.76
	16-44.9		1.05 (0.77-1.42)	0.76
	45+		1.11 (0.82-1.50)	0.52
Home care	Yes	<0.0001	0.74 (0.55-0.99)	0.04
	No		Reference	
Stage at diagnosis	Stage I/II/III		Reference	
	Stage IV	<0.0001	1.35 (1.08-1.68)	0.01
Orchiectomy	Yes	0.15	0.53 (0.20-1.40)	0.20
	No		Reference	
Castrate resistant	Yes	<0.0001	1.04 (0.83-1.31)	0.73
	No		Reference	
Prostatectomy after diagnosis	Yes	0.07	0.52 (0.35-0.76)	0.0009
	No		Reference	
Systemic therapy	LPT + bone-targeted Tx	<0.0001	1.86 (1.41-2.46)	<0.0001
	LPT alone		1.38 (1.05-1.82)	0.02
	Bone-targeted Tx alone		3.40 (2.10-5.51)	<0.0001
	None		Reference	
Number of hospitalizations	0		Reference	
	1-2	0.038	0.99 (0.72-1.36)	0.94
	3+		1.17 (0.84-1.64)	0.36

Patients had higher odds of receiving radiotherapy if they were registered at a designated regional cancer center (OR 1.8 [95% CI 1.3-2.4]). Furthermore, patients who were diagnosed more recently, between 2013 and 2018, were more likely to receive radiotherapy when compared with a prostate cancer diagnosis in 2007-2011 (odds ratio [OR] 1.3, 95% confidence interval [CI] 1.04-1.6).

Moreover, both radiation oncologist involvement (OR 155.1, 95% CI 83.3-288.7) and medical oncologist involvement (OR 1.4, 95% CI 1.1-1.8) were found to be associated with higher odds of receipt of radiation treatments when compared with no involvement.

In contrast, there was no significant association between the involvement of urologists in cancer care and receipt of radiotherapy (OR 1.2, 95% CI 0.9-1.6).

Odds of receipt of radiation treatments were higher in younger age groups (65-69 years) compared to those 85 years or older (OR 1.6, 95% CI 1.1-2.3; there was no statistically significant difference in other age groups).

Patients with stage IV disease at diagnosis were found to be more likely to have received radiation therapy, compared to patients with stages I, II, and III (OR 1.3, 95% CI 1.1-1.7). The odds of undergoing radiotherapy decreased in patients with five or more chronic conditions compared to 0 to 2 (OR 0.8, 95% CI 0.6-0.9). We found significant associations between receipt of systemic therapies and radiotherapy; patients who received LPT and bone-targeted treatments (OR 1.9, 95% CI 1.4-2.5), LPT alone (OR 1.4, 95% CI 1.05-1.8), or bone-targeted therapy alone (OR 3.4, 95% CI 2.01-5.5) were all more likely to receive radiotherapy.

There were no statistically significant associations between receipt of radiotherapy and relative income quintile within each neighborhood or LHIN region (except lower odds for a single LHIN, Table 2).

Likewise, distance to the nearest cancer center, home care involvement, the number of hospitalizations, a prior prostatectomy or orchiectomy, and Castrate Resistance Prostate Cancer (CRPC) status were not found to be associated with receipt of radiotherapy.

Radiotherapy trends before death

**Figure 1:**
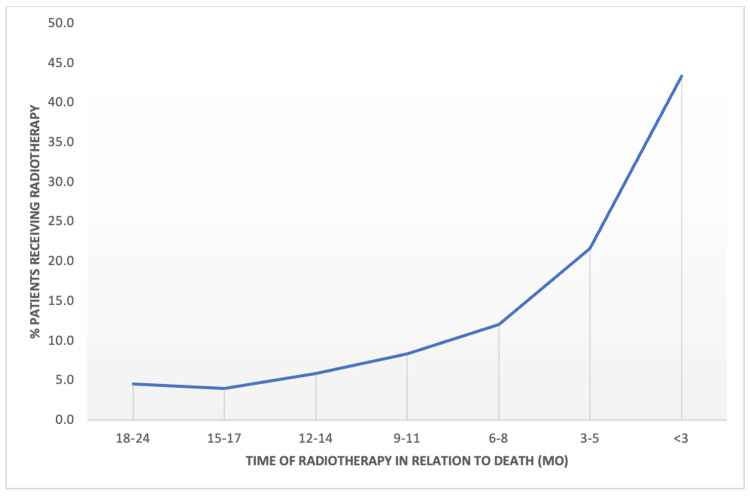
Proportion of patients receiving any radiotherapy before death, represented as a percentage of all patients in our cohort who received any radiotherapy in the last two years of life. Data are stratified by the number of months before death.

Analysis of the trends in the timing of radiation treatments in the last two years of life demonstrated that a higher proportion of patients received radiotherapy closer to death (Figure 1).

Increasingly more patients underwent radiotherapy in the last three months before death (*n *= 821, 43.4%) as compared with three to five months (*n *= 409, 21.6%), six to eight months (*n *= 229, 12.1%), 9-11 months (*n *= 158, 8.4%), 12-14 months (*n *= 111, 5.9%), 15-17 months (*n *= 75, 4.0%), and 18-24 months (*n *= 87, 4.6%).

## Discussion

In this large population-based study of prostate cancer decedents receiving ADT after diagnosis, we found that approximately half (1,890, 49.9%) of patients received palliative radiotherapy in the two years before death. This is in line with the target utilization rate of all radiotherapy services in the province. This target is set at 43% and determined using a criterion-based benchmarking approach; it measures the rate of any radiotherapy use (and not only palliative courses) in settings with little to no barriers to access, under the assumption that the rate of use of radiation services will be appropriate where there is optimal decision-making about the use and also unimpeded access to treatments [14,15]. Although we report on associations with receipt of any radiotherapy in this study, the results apply to palliative treatments. This is due to the specifics of cohort selection of prostate cancer decedents and the fact that curative intent therapy comprised merely 2.3% of all radiotherapy treatments in this investigation.

Patients diagnosed with stage IV at the outset were more likely to receive radiation treatment. This is in contrast to patients of all other stages combined at diagnosis, who later developed metastatic disease and became eligible for palliative radiotherapy. Also, the majority of patients received radiotherapy in the last three to six months of life. Palliative management of symptoms with other modalities other than radiotherapy, such as analgesics could be one of the reasons that not all metastatic patients require palliative radiotherapy. However, this could also be partly due to a lack of access to radiotherapy services or a lack of specialist care.

Patients with involvement of radiation and medical oncologists in their cancer care had a higher likelihood of undergoing radiation treatments, which is consistent with the need for regional cancer center registration (early on at diagnosis) that was identified to be a major factor associated with receipt of radiotherapy. This is supported by a review of the literature, suggesting that specialist cancer center care leads to improved outcomes and access to therapies [15-18]. Moreover, approximately 15% of Ontario’s population lives in rural geographic areas, and several investigations have highlighted the lack of ability to access oncologic care as a function of rural residency. For instance, in a recent study, 18.4% of patients with cancer in rural areas of Ontario did not have a consultation with a medical or radiation oncologist throughout their disease trajectory. Patients who were older or who resided in rural areas were significantly less likely to have had a consultation [15,19]. Although limited by access to high-speed internet, the increasing availability of virtual care may have the potential to improve access to specialist consultations for patients in remote areas or when in-person visits are deemed challenging. Future efforts should be focused on improving access to multidisciplinary regional cancer centers and also expanding technological infrastructures that could improve virtual access to specialist consultations.

While a greater distance to a radiotherapy center could be perceived as a significant barrier potentially resulting in decreased utilization of radiotherapy [20], our cohort did not show any association between distance to a cancer center and provincial healthcare region regarding the receipt of radiotherapy. Literature suggests that geographic location explains a significant proportion of the variation in the use of radiation to treat patients with nonmetastatic prostate cancer in the youngest and oldest age groups in private healthcare settings [21]. Likewise, despite universal access to health care in Canada, most radiotherapy centers are located in larger, more populated tertiary centers of southern Ontario, and a recent analysis has demonstrated suboptimal access to radiation treatment and poorer cancer outcomes for residents of the northern regions of the country as compared with southern parts [22]. In contrast to our study, patients with other malignancies with different radiotherapy indications could account for the differences in observed associations. Whereas travel time to the nearest radiotherapy center has been implicated in access to palliative radiotherapy for various cancers in other parts of Canada, the lack of association with distance to a cancer center in our study may be explained by the structure of cancer services between Ontario and other provinces [23,24,25,26].

There are several limitations to this study, including administrative databases as the only source of data, which do not include certain clinical details that may have been factored into the treatment decision-making process and subsequent receipt of radiotherapy. Moreover, given this population-based study is carried out in the context of Ontario's healthcare system, many factors could potentially contribute to the lack of generalizability of the findings to all patients and also regions with different healthcare structures. First, given the lack of drug benefit coverage in patients under the age of 65, and receipt of ADT as one of the main inclusion criteria, younger patients under the age of 65 were not included in the study. Furthermore, although we report on associations with receipt of any radiotherapy in this study, the results apply to palliative treatments. This is due to the specifics of cohort selection of prostate cancer decedents and the fact that curative intent therapy comprised merely 2.3% of all radiotherapy treatments in this investigation. Given the main focus of identifying barriers to access to radiotherapy services, the analysis included all patients (including the minority that received curative intent treatment) who received any radiotherapy. Moreover, all patients in this descendant cohort were included to be able to make references to subsequent studies using the same cohort of patients investigating the interplay between radiotherapy and novel androgen-targeted drugs. Also, a proportion of patients had missing data in one or more variables (including OHIP billing data through which involvement of oncologists was determined) and were excluded from the statistical analyses with regression models, thus potentially introducing an information bias. Furthermore, income levels were classified into quintiles within each neighborhood, and they are considered relative measures of income disparity within each neighborhood and dependent on the average income level within each district.

## Conclusions

In conclusion, this population-based study of prostate cancer decedents is the first of its kind to demonstrate a positive association with receipt of radiotherapy and younger age, metastatic disease at diagnosis, fewer comorbidities, patient registration at regional cancer centers, and the involvement of oncologists. Given all patients had universal access to healthcare and drug benefits, there were no differences detected based on income or distance from a cancer center with receiving radiation treatments. Since unimpeded access to radiation therapy continues to be a challenge, future efforts should be focused on improving access to multidisciplinary regional cancer centers to improve the quality of life of many metastatic patients.

## Figures and Tables

**Figure 2:**
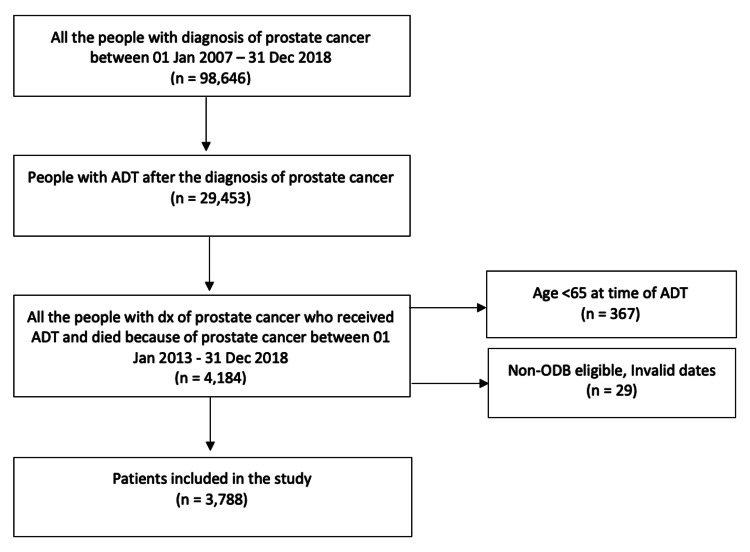
Data flow diagram with inclusion and exclusion criteria used to construct the patient cohort. ADT, androgen deprivation therapy; ODB, Ontario Drug Benefit

## Appendices

Appendix A 

**Table 5:** Databases utilized to collect study variables. This table outlines the databases hosted under Institute for Clinical Evaluative Sciences (ICES) that were used to collect the data included in this study, accompanied by a brief description of information gathered from that database.

Database	Description
Ontario Drug Benefit (ODB)	Captures all ODB drug claims by patients and was utilized to identify receipt of drugs funded by this program.
Ontario Cancer Registry (OCR)	Provincial database of information for all those diagnosed with cancer. The date and diagnosis of prostate cancer and year of death were captured utilizing this.
Discharge Abstract Database (DAD)	Captures all acute care use. This was utilized to help capture those who underwent bilateral orchiectomy, admission to a palliative care unit, and metastatic status within 6 months of diagnosis.
Registered Persons Database (RPDB)	Utilized to capture demographic data including age, sex, and postal code.
Ontario Health Insurance Plan Claims Database (OHIP)	Utilized to capture all claims for physician services, including physician involvement, procedures performed, and number of visits to them during the study period.
National Ambulatory Care Reporting System (NACRS)	Captured all provincial emergency department encounters.
Cancer Activity Level Reporting (ALR)	Captures activity within the cancer system such as treatment sessions and clinic visits, and was utilized to determine regional cancer center registration and receipt of some systemic therapies and radiotherapy.
Client Agency Program Enrolment (CAPE)	Captures information on a patient’s association with a primary care physician or care team, and was utilized to determine whether patients were rostered to primary care.
Continuing Care Reporting System (CCRS)	Captured whether a patient is a long-term care resident.
Ontario Census Area Profiles (CENSUS)	Captured rurality and income quintiles using patient postal codes.
Postal Code Conversion File (PCCF)	Linked six-character postal codes and standard census data for that geographic area.
ICES Physician Database (IPDB)	Utilized to capture physician specialty.
New Drug Funding Program (NDFP)	Captured cancer drugs administered in hospitals or cancer centers that are funded by this program.
Ontario Laboratories Information System (OLIS)	Captured laboratory data.
Home Care Database (HCD)	Captured whether any home care services were delivered to patients.
Information about Ontario healthcare institutions funded by the Ministry of Health and Long-Term Care (INST)	Captured data about all publicly funded healthcare institutions.

Appendix B
